# Cost-effectiveness of a telephone health coaching and remote exercise monitoring for peripheral artery disease (TeGeCoach)^☆^

**DOI:** 10.1016/j.ajpc.2026.101746

**Published:** 2026-07-14

**Authors:** Dirk Heider, Farhad Rezvani, Herbert Matschinger, Jörg Dirmaier, Martin Härter, Lutz Herbarth, Patrick Steinisch, Gundula Krack, Thomas Korth, Lara Thomsen, Daniela Patricia Chase, Robert Schreiber, Mark-Dominik Alscher, Benjamin Finger, Hans-Helmut König

**Affiliations:** a Department of Health Economics and Health Services Research, University Medical Center Hamburg-Eppendorf, Hamburg, Germany; b Department of Medical Psychology, University Medical Center Hamburg-Eppendorf, Hamburg, Germany; c KKH Kaufmännische Krankenkasse statutory health insurance, Hannover, Germany; d mhplus Krankenkasse statutory health insurance, Ludwigsburg, Germany; e IEM GmbH, Aachen, Germany; f Philips GmbH, Hamburg, Germany; g Robert Bosch Gesellschaft für medizinische Forschung mbH, Dr. Margarete Fischer-Bosch-Institute of Clinical Pharmacology, Stuttgart, Germany

**Keywords:** Peripheral arterial disease, Randomized-controlled clinical trial, Home-based exercise program, Cost-effectiveness, Intention-to-treat, Modified intention-to-treat, Per protocol

## Abstract

Introduction The number of people with peripheral arterial disease (PAD) increased by almost 25% between 2000 and 2010. The economic burden on healthcare systems caused by PAD will continue to rise because of the aging population and a further increase in risk factors. The aim of the study was to examine the cost-effectiveness of the 12-month patient-centered ‘Telephone Health Coaching and Remote Exercise Monitoring for Peripheral Artery Disease’ (TeGeCoach) program in a randomized controlled trial. Methods This is a cost-effectiveness analysis based on a two-arm, parallel-group, open-label, pragmatic, randomized-controlled clinical trial conducted by three German statutory health insurance funds with follow-up assessments after 12 and 24 months. Claims data for the calculation of costs and questionnaire data for the quantification of quality-adjusted life years (QALYs) based on the EQ5D-5L were used. From a healthcare payer’s perspective, cost-effectiveness was examined over 24 months follow-up using intention-to-treat (ITT) and modified ITT (mITT) and per protocol (PP) approaches. Results Assuming intervention costs of €672, the point estimates for the ITT analysis over 24 months showed non-significantly higher costs in the IG of €2003 and non-significantly higher QALYs of 0.02 compared to the control group. At a willingness to pay (WTP) threshold of €50,000, the probability of the TeGeCoach being cost-effective was 30%. Under the same assumptions, the mITT and the PP approach resulted in probabilities for cost-effectiveness of 79% and, 95%, respectively. Conclusion As demonstrated by the PP- and mITT-analysis, the treatment of PAD by TeGeCoach might be cost-effective under favorable assumptions. However, applying the stricter criteria represented by the ITT scenario, TeGeCoach couldn’t demonstrate its cost-effectiveness. Therefore, before TeGeCoach should be used in healthcare practice, it is necessary to adapt the program implementation. The effectiveness of these adjustments should then be verified in further studies.

## 1 Introduction

Affecting over 200 million people worldwide, peripheral arterial disease (PAD) is the third most common atherosclerotic cardiovascular disease. Especially in older adults PAD is a leading cause of disability and death [1,2]. In 2016, PAD costs were estimated at €5552 per patient in a French study [3]. Costs increase with the severity of PAD. In the US, Fontaine stages 1 and 2 caused total annual healthcare costs of $2.8 billion in 2014, while Fontaine stages 3 and 4 caused costs of $3.5 billion [4].

Supervised exercise programs (SEPs) for patients with PAD are limited by financial and organizational barriers. When SEPs cannot be provided, structured home-based exercise programs (HEPs) are an alternative and are recommended as second-line therapy, improving walking impairment, though results vary.

The TeGeCoach study evaluated a 12-month, telemonitored, motivational HEP with wearable activity tracking [[5], [6], [7]]. TeGeCoach differs from other HEPs primarily in its longer duration and more intensive support of PAD patients. The aim of the present study was to analyze the cost-effectiveness of TeGeCoach by calculating health care costs from health insurance claims data and quality-adjusted life years (QALYs) based on the participants’ ratings on the EQ5D-5L for a 24-month follow-up period.

## 2 Methods

### 2.1 Study participants

The TeGeCoach study was a two-arm, parallel-group, open-label, pragmatic, randomized-controlled clinical trial. TeGeCoach was administered by three German statutory health insurance funds (KKH Kaufmännische Krankenkasse, TK Techniker Krankenkasse, and mhplus Krankenkasse). Inclusion criteria were: being insured at one of the three participating statutory health insurance funds; aged between 35 and 80; German-speaking; access to a telephone (landline or mobile); and a primary or secondary diagnosis of PAD at Fontaine stage IIa or IIb within the last 36 months. The participating statutory health insurance funds selected and contacted potential participants.

Ethical approval was obtained from the ethics committee of the Medical Association Hamburg (Ärztekammer Hamburg). TeGeCoach has been registered at www.clinicaltrials.gov (NCT03496948), and protocol modifications were added to the trial registry.

### 2.2 Intervention

TeGeCoach, a 12-month patient-centered HEP, aims to improve PAD outcomes by inspiring healthy habits and changing unhealthy ones. Participants wore activity trackers to self-monitor their steps, and data was automatically transferred to telemedicine centers for feedback from coaches. Walking plans were assigned based on functional status and exercise capacity: 15, 15–30, or 60 min daily. Participants walked to maximal tolerable pain with rests, following interval training principles. Along with the walking exercise, patient-tailored topics relevant to PAD management were discussed to improve health literacy. Participants had up to nine structured 30–60-min phone calls with coaches to discuss progress, review data, and check adherence. In cases of poor adherence, additional phone calls and supportive informational materials were provided. During additional 12 months of unstructured follow-up, participants were able continue wearing their activity trackers.

### 2.3 Study outcomes

At baseline, after 12 and 24 months, the EQ5D index score was calculated based on the EQ5D-5L questionnaire using the German value set. QALYs were calculated for the entire 24-month follow-up period using the area under the curve method. Using health insurance claims data, health care costs from the perspective of the health insurance were measured over the 24-month follow-up period in terms of costs for outpatient physician services, outpatient non-physician services, medical supplies, inpatient treatment, sick pay, medication, rehabilitation and prevention training. All costs in these nine categories were summed to obtain a variable that represented total costs. Including costs for health coaches, fitness trackers, software, and information packets, intervention costs were estimated at €672 in accordance with accounting principles.

### 2.4 Statistical analysis

The cost-effectiveness was analyzed using the intention-to-treat (ITT) and per-protocol (PP) approach. The ITT-population included participants who had completed a questionnaire at baseline. The PP population comprised of participants who had completed nine coaching calls. Missing data due to drop-out, missing individual assessments, or missing information in individual items of the EQ5D were imputed for each scenario. By using multiple imputation by chained equation (MICE) forty imputed datasets were created with predictive mean matching (PMM). For the 24-month follow-up, mean costs and effects were calculated for the IC and CG. Cost-effectiveness planes were used for graphically presentation of the results. Cost-effectiveness acceptance curves (CEAC) were estimated based on the seemingly unrelated regression analyses and bootstrapping. All analyses were conducted using STATA/MP 19.

## 3 Results

### 3.1 Patient characteristics

The ITT-sample comprised 1685 participants with 68.3% being male. The average age was 66.5 years. 62.8% were insured with TK, 34.5% with KKH, and 2.7% with mhplus. 60.5% were pensioners, 37% were regularly insured, and 2.4% were insured as family members. There were no notable differences between IC and CG with respect to baseline sample characteristics (Fig. 1A). The PP-sample comprised of 1370 participants.

**Fig. 1 Panel A:**
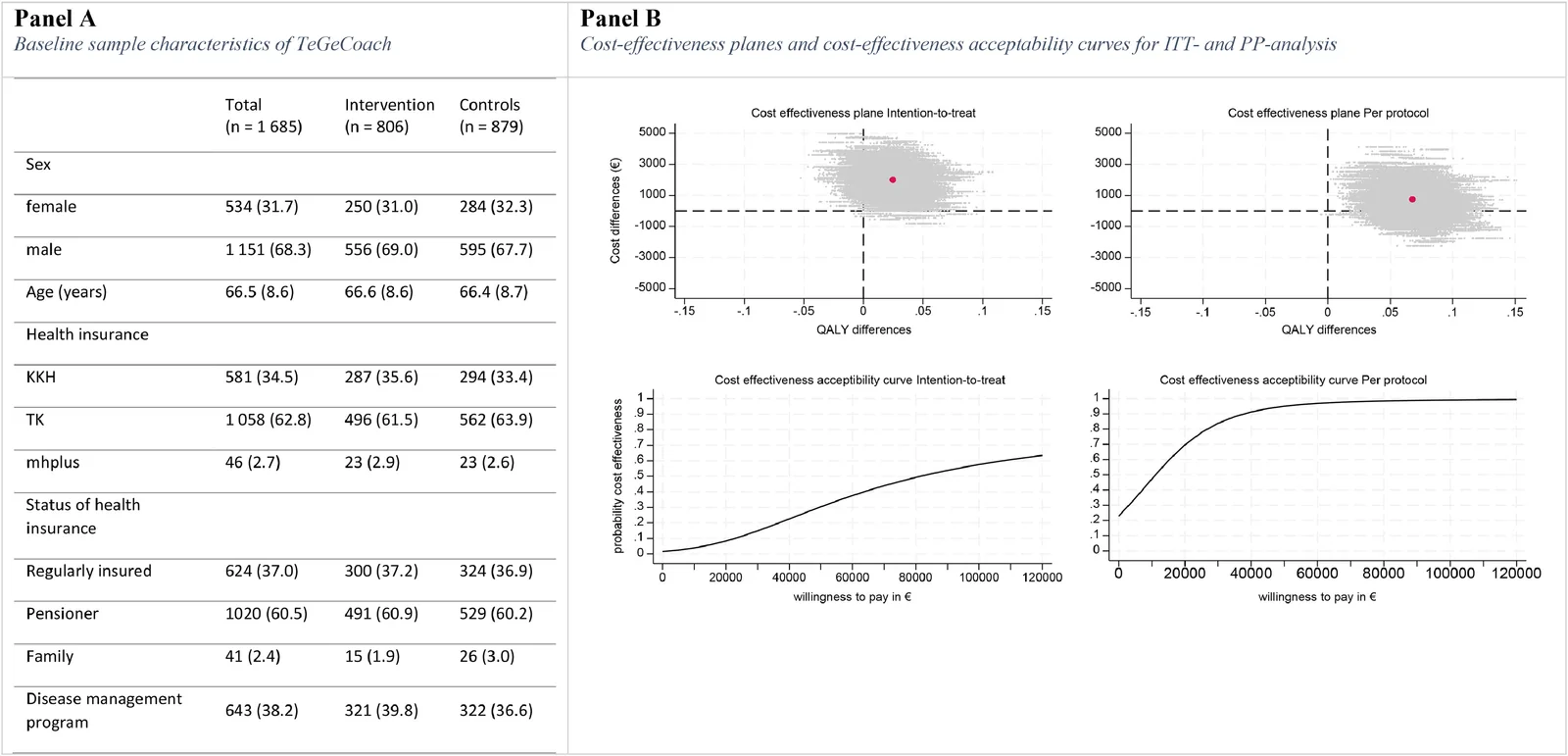
Baseline sample characteristics on socio-demographics and health insurance status of the randomized participants for ITT sample in TeGeCoach. All values are expressed as means and standard deviations.

### 3.2 ITT analysis

The average total costs over the 24-month follow-up period amounted to €14,957 in the IG and €12,954 in the CG. The costs for hospital treatment were €6852 in the IG and €5969 in the CG. Costs for outpatient physician services were nearly identical in both groups with €2661 in the IG and €2689 in the CG. Costs for medications were €2588 in the IG and €2453 in the CG. Costs for sick pay were €896 in the IG and €623 in the CG. Only the difference of €2003 on total costs was significant (*p* < 0.05). Considering the insignificant difference in QALYs of 0.02, the ICER was 81,479 €/QALY. The point estimate is located in the North-East Quadrant of the cost-effectiveness plane (Fig. 1B), indicating the IG being more effective but more costly than the CG. Assuming a hypothetical willingness to pay (WTP) of €50,000 per QALY, the CEAC for the TeGeCoach intervention in Fig. 1B shows probability for cost-effectiveness of 30%.

**Fig. 1. Panel B:**
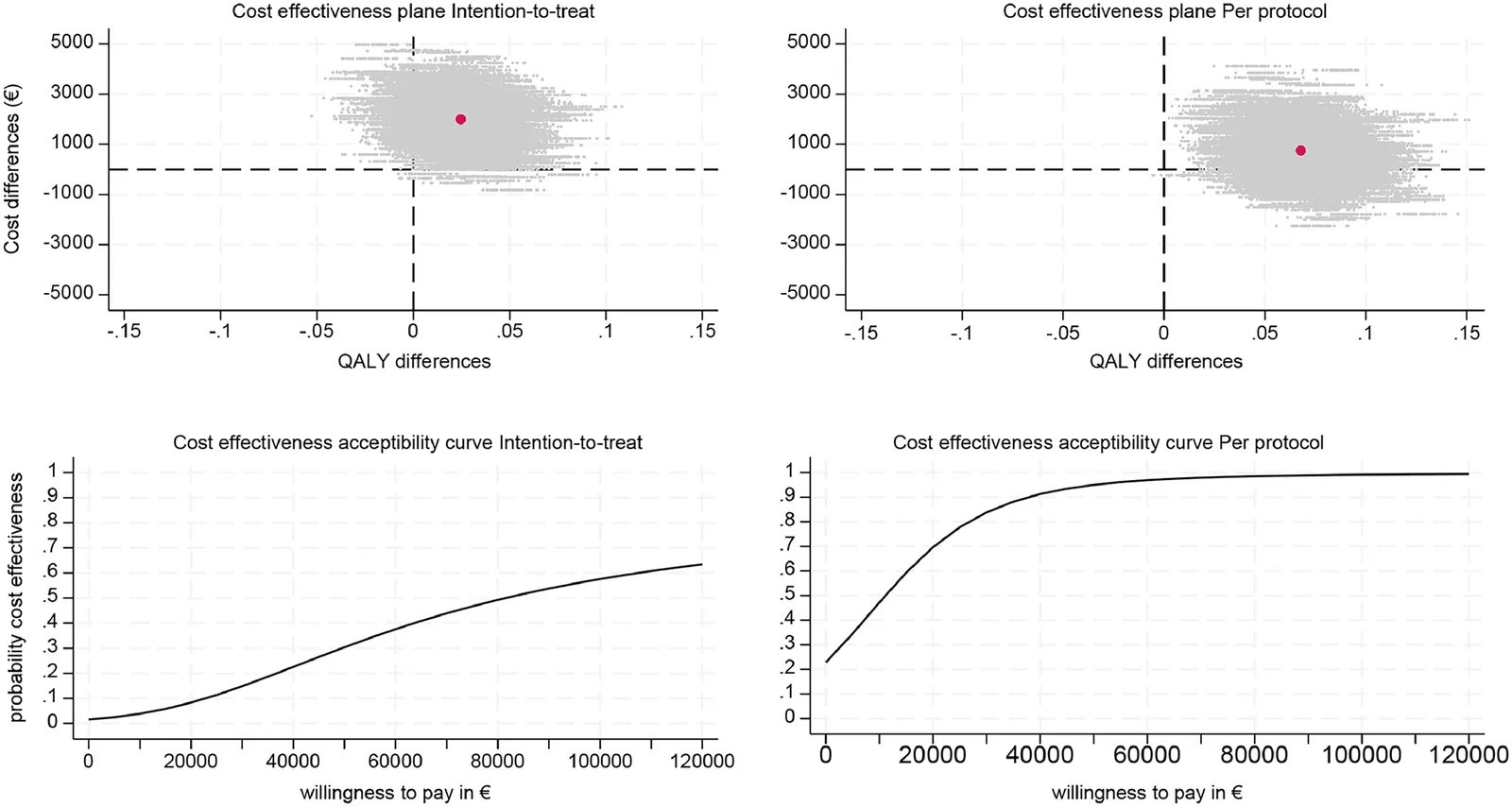
Upper row: Cost-effectiveness planes for TeGeCoach intervention with 24-month follow-up. Lower row: Cost-effectiveness acceptability curves for TeGeCoach with 24-month follow-up. Left column: ITT analysis, right column: PP analysis.

### 3.3 Per-protocol analysis

For PP the significant effect on QALYs was 0.07 (*p* < 0.01). With €753, the insignificant difference in mean total costs was lower than for the ITT analysis resulting in an ICER of 11,106 €/QALY. Assuming a hypothetical WTP of €50,000 per QALY, the CEAC shows probabilities for cost-effectiveness of 95% for the PP-analyses (Fig. 1B).

## 4 Discussion

As demonstrated by the PP-analysis, the treatment of PAD by TeGeCoach might be cost-effective under favorable assumptions. However, applying the stricter criteria represented by the ITT scenario, TeGeCoach was not cost-effective. The results of the PP analysis would be relevant, if a potential provider of TeGeCoach could significantly reduce dropout rates, for example, by selecting specific target groups. A general reduction in the dropout rate would require considerable additional efforts to keep participants engaged. With nine coaching calls for the PP population, these efforts were already very high. Particularly in routine care, this makes further improvements in patient adherence rather unlikely. Alternatively, it might be promising to assign patients to specialists like angiologists as non-assignment to a GP was a major factor preventing patients from starting the program. Therefore, before TeGeCoach should be used in healthcare practice, it is necessary to adapt the program implementation. The effectiveness of these adjustments should then be verified in further studies. Strengths of the study are its large sample size, broad inclusion criteria, long-term participant follow-up and flexible adherence protocols applicable to everyday clinical practice ensuring the generalizability of the study results. Restricting the generalizability of the findings, the low enrolment rate (3.1%) is a limitation.

## Funding

This clinical trial receives funding from the German Innovation Fund (01NVF17013) of the Federal Joint Federal Committee (G-BA), the highest decision-making body of the joint self-government of physicians, dentists, hospitals and health insurance funds in Germany. The use of grant funds is monitored by the German Aerospace Center (Deutsches Zentrum für Luft- und Raumfahrt; DLR). Neither the G-BA nor the DLR will be involved in the actual conduct of this work (i.e. execution, data analysis, interpretation of data, dissemination).

## CRediT authorship contribution statement

**Dirk Heider:** Writing – original draft, Methodology, Formal analysis. **Farhad Rezvani:** Writing – review & editing, Project administration. **Herbert Matschinger:** Writing – review & editing. **Jörg Dirmaier:** Writing – review & editing. **Martin Härter:** Writing – review & editing, Supervision. **Lutz Herbarth:** Writing – review & editing. **Patrick Steinisch:** Writing – review & editing. **Gundula Krack:** Writing – review & editing. **Thomas Korth:** Writing – review & editing. **Lara Thomsen:** Writing – review & editing. **Daniela Patricia Chase:** Writing – review & editing. **Robert Schreiber:** Writing – review & editing. **Mark-Dominik Alscher:** Writing – review & editing. **Benjamin Finger:** Writing – review & editing. **Hans-Helmut König:** Writing – review & editing, Supervision, Conceptualization.

## Declaration of competing interest

The authors declare that they have no known competing financial interests or personal relationships that could have appeared to influence the work reported in this paper.
